# Frailty status among older critically ill patients with severe acute kidney injury

**DOI:** 10.1186/s13054-021-03510-y

**Published:** 2021-02-25

**Authors:** William Beaubien-Souligny, Alan Yang, Gerald Lebovic, Ron Wald, Sean M. Bagshaw

**Affiliations:** 1 Division of Nephrology, Centre Hospitalier de L’Université de Montréal, Montreal, Canada; 2 Applied Health Research Centre, St. Michael’s Hospital, Toronto, Canada; 3 Division of Nephrology, St. Michael’s Hospital and University of Toronto, Toronto, Canada; 4 Department of Critical Care Medicine, Faculty of Medicine and Dentistry, School of Public Health, University of Alberta, 2-124 Clinical Science Building, 8440-112 Street, Edmonton, AB T6G2B7 Canada

**Keywords:** Acute kidney injury, Frailty, Renal replacement therapy, Patient-oriented outcomes, Aging, Functional status, Quality of life

## Abstract

**Background:**

Frailty status among critically ill patients with acute kidney injury (AKI) is not well described despite its importance for prognostication and informed decision-making on life-sustaining therapies. In this study, we aim to describe the epidemiology of frailty in a cohort of older critically ill patients with severe AKI, the outcomes of patients with pre-existing frailty before AKI and the factors associated with a worsening frailty status among survivors.

**Methods:**

This was a secondary analysis of a prospective multicentre observational study that enrolled older (age > 65 years) critically ill patients with AKI. The clinical frailty scale (CFS) score was captured at baseline, at 6 months and at 12 months among survivors. Frailty was defined as a CFS score of ≥ 5. Demographic, clinical and physiological variables associated with frailty as baseline were described. Multivariable Cox proportional hazard models were constructed to describe the association between frailty and 90-day mortality. Demographic and clinical factors associated with worsening frailty status at 6 months and 12 months were described using multivariable logistic regression analysis and multistate models.

**Results:**

Among the 462 patients in our cohort, median (IQR) baseline CFS score was 4 (3–5), with 141 (31%) patients considered frail. Pre-existing frailty was associated with greater hazard of 90-day mortality (59% (*n* = 83) for frail vs. 31% (*n* = 100) for non-frail; adjusted hazards ratio [HR] 1.49; 95% CI 1.11–2.01, *p* = 0.008). At 6 months, 68 patients (28% of survivors) were frail. Of these, 57% (*n* = 39) were not classified as frail at baseline. Between 6 and 12 months of follow-up, 9 (4% of survivors) patients transitioned from a frail to a not frail status while 10 (4% of survivors) patients became frail and 11 (5% of survivors) patients died. In multivariable analysis, age was independently associated with worsening CFS score from baseline to 6 months (adjusted odds ratio [OR] 1.08; 95% CI 1.03–1.13, *p* = 0.003).

**Conclusions:**

Pre-existing frailty is an independent risk factor for mortality among older critically ill patients with severe AKI. A substantial proportion of survivors experience declining function and worsened frailty status within one year.

## Background

Severe acute kidney injury (AKI) occurs in a significant proportion of critically ill patients. Its presence is a marker of the severity of critical illness, and therefore, severe AKI is often associated with organ dysfunction and with adverse outcomes [1]. Despite important advances in understanding the epidemiology of this condition, the long-term outcomes among survivors of severe AKI are more limited. There is a dearth of information on patient-centred concerns such as functional status, quality of life and socioeconomic status in this population.

A deterioration in health-related quality of life is known to occur in a significant proportion of patients after critical illness, whether they develop AKI or not [2, 3]. However, the presence of severe AKI might herald a higher risk of physical limitations and disability [2]. Frailty is a state of vulnerability characterized by a diminished resilience to external stressors [4] and has been shown to portend greater risk of adverse outcomes in critical illness [5–7]. Notably, a frail state before critical illness is associated with worse functional status and health-related quality of life among survivors during follow-up [8].

Recent data have suggested AKI may be associated with greater declines in functional status and frailty among survivors of critical illness at 12 months compared to those without AKI [9]. However, there has been no description of how frailty status may evolve among survivors whose course was complicated by severe AKI and whether there are identifiable factors associated with clinical deterioration. Accordingly, the primary aim of this study was to describe frailty status in a prospective cohort of older critically ill patients with severe AKI at 6 months and 12 months. As secondary objectives, we further aimed to describe the association of baseline frailty status and 90-day mortality and identify factors associated with deterioration in frailty status at follow-up.

## Methods

### Design, setting, and participants

We conducted a secondary analysis of the Optimal Selection for and Timing to Start Renal Replacement in Critically Ill Older Patients with Acute Kidney Injury (OPTIMAL-AKI), a multicentre prospective cohort study performed at 16 hospitals across Canada between September 23, 2013 and November 18, 2015 [10]. Approval for the study was received by the Research Ethics Board at the University of Alberta (File Pro00037850) and each participating centre. All participants or their legally authorized surrogate provided informed consent. This study follows the recommended reporting outlined in the strengthening the reporting of observational studies in epidemiology (STROBE) Statement [11].

OPTIMAL-AKI included patients ≥ 65 years old admitted to the intensive care unit (ICU) with severe AKI, defined as either: (1) a threefold increase in serum creatinine from a known premorbid baseline or during the current hospitalization, (2) a serum creatinine > 4.0 mg/dl (> 354 μmol/L) with evidence of a minimum increase of 0.3 mg/dl (27 μmol/L), (3) urine output < 7.2 ml/kg during the past 24 h, (4) complete anuria for the preceding 12 h or (5) twofold increase in serum creatinine from a known premorbid baseline or during the current hospitalization and total urine output < 6.0 ml/kg over the preceding 12 h (or < 2 ml/kg over 4 h). Patients who received renal replacement therapy (RRT) in the preceding 4 weeks or with drug intoxication representing an indication of urgent RRT initiation were excluded. For the purpose of this analysis, patients without frailty status data recorded at baseline were excluded.

### Data collection

Trained research coordinators performed standardized data collection using a defined protocol, as originally described [10]. Pre-hospital frailty status was assessed using the 9-point clinical frailty scale (CFS) score [4]. However, it was pre-specified that patients could not be assigned a score of 9 (terminally ill). Trained coordinators captured the CFS using multiple data sources including the medical record and a baseline interview with the patient and/or family members. This assessment was previously demonstrated to be reliable in the ICU setting [12]. Patients and/or family members were contacted at 6-months and 12-months to ascertain vital status, disposition, and frailty status.

### Definitions and outcomes

Our primary objective was to describe the trajectory of frailty from baseline through 12 months of follow-up. We defined frailty as a CFS score of ≥ 5 [4, 13]. Our secondary objective was to evaluate the association between baseline frailty status and 90-day all-cause mortality [10]. We also evaluated the incidence of new onset of frailty and deterioration of frailty status during follow-up at 6 months and 12 months [4, 13]. New onset frailty was defined as the transition from a baseline CFS score < 5 to a CFS score ≥ 5. Deterioration of frailty status was defined as a transition from a lower category of frailty status at baseline to a higher category. Frailty was categorically graded by stratifying CFS scores of 1–3 as fit; scores of 4 as vulnerable; scores of 5 as mildly frail; and 6–8 as moderate to severely frail. These categories have been used previously in the Canadian elderly population [4, 7].

### Statistical analysis

We examined characteristics of subjects stratified by CFS (both binary and categorical classifications) at baseline. For continuous variables the association was tested using a t-test (or an Analysis of Variance F-test for categorical classification of CFS) and for categorical variables the association was tested using either Pearson’s Chi-square test or Fisher’s Exact test, as appropriate. Kaplan–Meier survival curves were created to display the natural history of all-cause mortality by CFS for up to 12 months of follow-up. Log-rank tests were used to test the difference in survival probability based on frailty status. The normality assumption for continuous variables was assessed through visual inspection of Normal Q-Q plots. In the presence of serious non-normality, the nonparametric Kruskal–Wallis rank sum test was used. The homogeneity of variance assumption was assessed through the Levene’s test for equality of variances.

To determine the association between frailty and 90-day mortality, we employed Cox proportional hazards regression for both unadjusted and adjusted analyses. In the Cox proportional hazards models, we evaluated the association between CFS and mortality, censored at date of last follow-up or at the end point of the time of interest. The covariates in the adjusted models included age, sex, Charlson Comorbidity Index (CCI) score, baseline eGFR, peak serum creatinine during current ICU admission and SOFA score and APACHE II score. These were a priori selected patient-level factors, physician-specific and site-specific factors or those deemed clinically important. The proportional hazards assumption was tested using visual inspection and statistical testing of the models Schoenfeld residuals. Model estimates and results from complete cases from the original dataset and using multiple imputed datasets were compared.

For variables used in multivariable models other than frailty and mortality, multiple imputations were used to impute the missing data. Different techniques were used according to variable type [14]. Predictive mean matching was used to impute continuous variables [15], logistic regression imputation was used for binary variables and polytomous regression imputation for unordered categorical data [14]. The set of variables that were used in the multiple imputation processes were the covariates in the adjusted models in the confirmatory analyses and other clinically important variables. The dataset used in the confirmatory analyses has roughly 10% missing data; thus, the imputation process was repeated 10 times which in turn produced 10 imputed datasets at random. The model estimates from the 10 datasets were then pooled to get final bias-adjusted estimates.

Multivariable prediction models were created using logistic regression with the same covariates. The performance of the model was assessed by evaluating the discrimination by the area under the receiver-operator curve (AUC) while calibration was assessed graphically. The Net Reclassification Improvement (NRI) index produced by the addition of the frailty information to a model containing the other baseline variables was computed.

To determine the predictors of a worsening CFS score from baseline to 6 months and subsequently to 12 months in survivors, we first performed univariable logistic regression to determine the association with clinically important variables including age, CCI score, APACHE II score, pre-hospital disposition, baseline CFS score, receipt of RRT, hospital and ICU length of stay. Patients who were already classified as moderately or severely frail at baseline, and thus could not get worse, were excluded from this analysis.

Additionally, we performed a multistate Markov model to determine the association between clinical variables and the transition between one of the following states to another: alive and not frail; alive and frail; and dead [16, 17].

All results are presented with a 95% confidence interval (CI) and a two-sided p-value of < 0.05 was considered significant. All statistical analyses were conducted in R (R Development team, 2010).

## Results

**Fig. 1:**
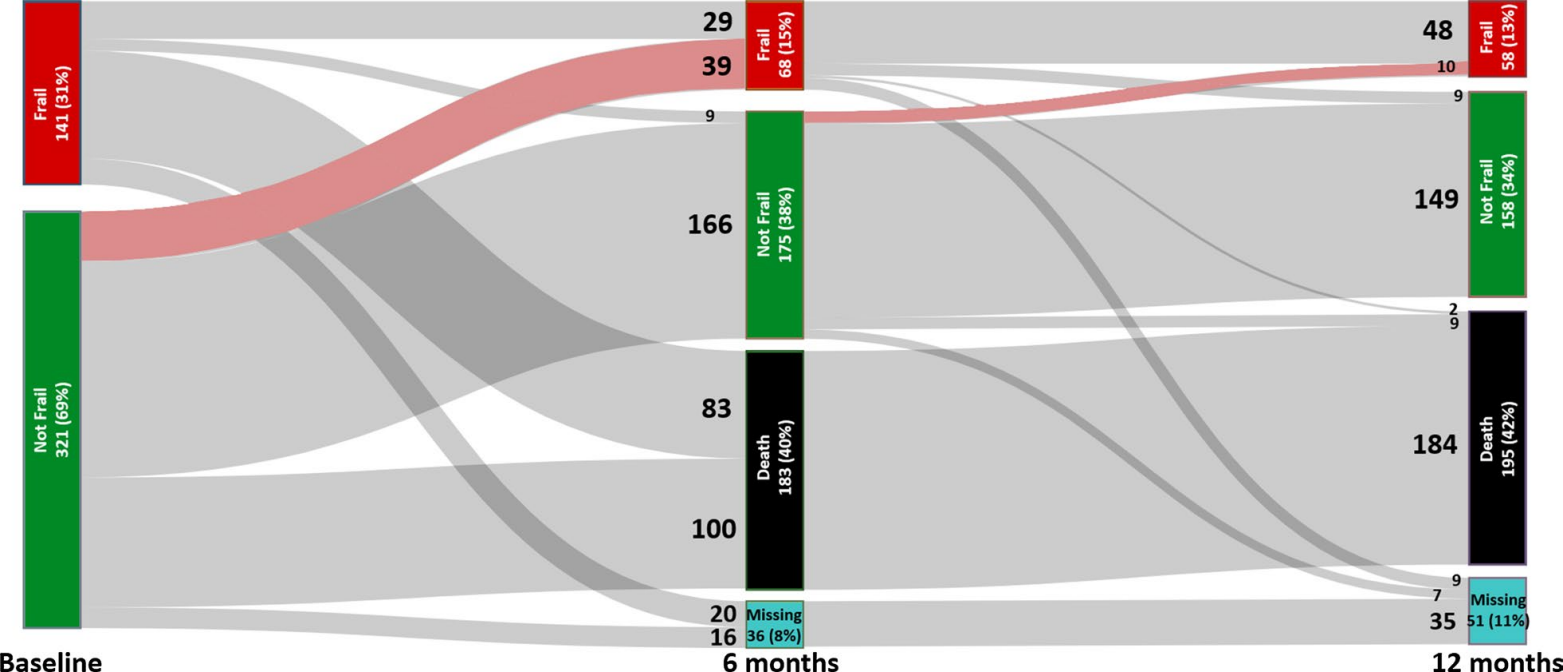
Sankey diagram showing frailty status in 462 patients with available CFS score at baseline in the OPTIMAL-AKI cohort. Links in pink are proportional to the number of patients transitioning to a frail status

Among the 499 patients enrolled in the OPTIMAL-AKI study, 462 (93%) had baseline CFS available (median score 4 [IQR 3–5]) and 141 (31%) patients were considered frail. Frailty data were available for 243 patients at 6 months (87% of surviving patients) and 216 patients at 12 months (81% of surviving patients) (Additional file 1: Figure S1). The complete distribution of CFS scores in the cohort is presented in Additional file 1: Table S1. During the 12-month follow-up period, 195 (42%) patients died and 51 (11%) were lost to follow-up. The evolution of frailty status from baseline to 12 months after enrolment is presented in Fig. 1.

### Pre-existing frailty status

Patient characteristics stratified by baseline frailty status are presented in Table 1. Patients with frailty were older, more likely to be female and had more comorbid disease. Patients with frailty were also more likely to have baseline cognitive impairment, to require assistance at home or reside in an assisted living facility and to have been hospitalized in the preceding 6 months. Clinicians were less willing to offer RRT to patients with frailty compared to those not frail (63.1% vs 76.0%, OR 0.83; 95% CI 0.72–0.96, *p* = 0.0067, and frail patients were subsequently less likely to receive RRT (37.6% vs 51.4%, OR 0.73; 95% CI 0.58–0.93, *p* = 0.0064). Patient characteristics stratified by category of frailty score are presented in Additional file 1: Table S2.

**Table 1:** Summary of patient characteristics stratified by frailty status at baseline

Variables	Frail 141(30.5%)	Not frail 321 (69.5%)	*p* value	Missing values (%)
*Demographic information*				
Age (mean [SD]), years	77.4 (8.1)	74.3 (6.7)	< 0.001	0.0
Female sex, (*n*, %)	74 (52.5)	114 (35.5)	0.001	0.0
Charlson score, (median [IQR])	2.0 (1.0, 4.0)	3.0 (2.0, 5.0)	< 0.001	0.2
*Comorbid diseases, (n, %)*				
Congestive heart failure	44 (31.2)	67 (20.9)	0.024	0.2
Chronic obstructive pulmonary disease	46 (32.6)	85 (26.6)	0.223	0.2
Connective tissue disease	11 (7.8)	16 (5.0)	0.335	0.2
Diabetes mellitus	59 (41.8)	123 (38.4)	0.558	0.2
Peripheral vascular disease	30 (21.3)	41 (12.8)	0.029	0.2
Any cancer	26 (18.4)	63 (19.7)	0.853	0.2
Chronic liver disease	8 (5.7)	8 (2.5)	0.150	0.2
Functional status				
Cognition impairment, (*n*, %)				
Dementia	18 (13.8)	2 (0.7)		
Impaired—not demented	44 (33.8)	28 (9.5)	< 0.001	13.0
No impairment	68 (52.3)	266 (89.9)		
Pre-hospital location				
Home—independent	45 (32.1)	283 (88.4)		
Home—with assistance	72 (51.4)	29 (9.1)	< 0.001	2.8
Assisted living	23 (16.4)	8 (2.5)		
Hospitalized in prior 6 months, (*n*, %)	66 (46.8)	110 (34.3)	0.014	1.8
Current illness				
Primary diagnostic category, (*n*, %)				
Cardiovascular	38 (27.0)	83 (26.2)		
Respiratory	29 (20.6)	64 (20.2)		
Gastrointestinal/hepatic	12 (8.5)	42 (13.2)		
Metabolic/endocrine	11 (7.8)	28 (8.8)	0.068	0.8
Neurologic	5 (3.5)	3 (0.9)		
Hematologic/oncologic	0 (0.0)	8 (2.5)		
Sepsis	45 (31.9)	79 (24.9)		
Trauma	1 (0.7)	10 (3.2)		
APACHE II score (mean [SD])	27.8 (8.7)	28.4 (8.8)	0.513	3.6
SOFA score (mean [SD])	9.8 (4.0)	10.9 (4.3)	0.011	4.0
Mechanical ventilation (*n*, %)	87 (63.5)	206 (66.7)	0.588	3.4
Vasoactive support (*n*, %)	89 (65.0)	212 (68.6)	0.517	3.4
Blood transfusion (*n*, %)	17 (12.4)	64 (20.7)	0.049	3.4
Total parenteral nutrition (*n*, %)	4 (2.9)	16 (5.2)	0.415	3.4
Baseline serum creatinine, (median [IQR])	97.0 (75.0, 133.0)	95.0 (72.0, 141.0)	0.972	0.6
Baseline eGFR, (mean [SD])	56.1 (31.7)	55.7 (30.9)	0.888	0.6
Worst KDIGO AKI stage, (*n*, %)				
Stage 2	28 (19.9)	58 (18.1)	0.756	0.2
Stage 3	113 (80.1)	262 (81.9)		
Receipt of RRT (*n*, %)	53 (37.6)	165 (51.4)	0.006	0.0
Peak BUN, (median [IQR])	24.9 (16.5, 33.3)	27.9 (18.9, 38.0)	0.034	2.4
Peak serum creatinine, (median [IQR])	305.0 (208.0, 442.0)	354.0 (242.0, 493.0)	0.006	0.0

AKI: acute kidney injury, APACHE II: acute physiology and chronic health evaluation II, eGFR: estimated glomerular filtration rate, KDIGO: acute physiology and chronic health evaluation II SD: standard deviation, SOFA: sequential organ failure assessment, RRT: renal replacement therapy

### Mortality at 90 days

**Fig. 2:**
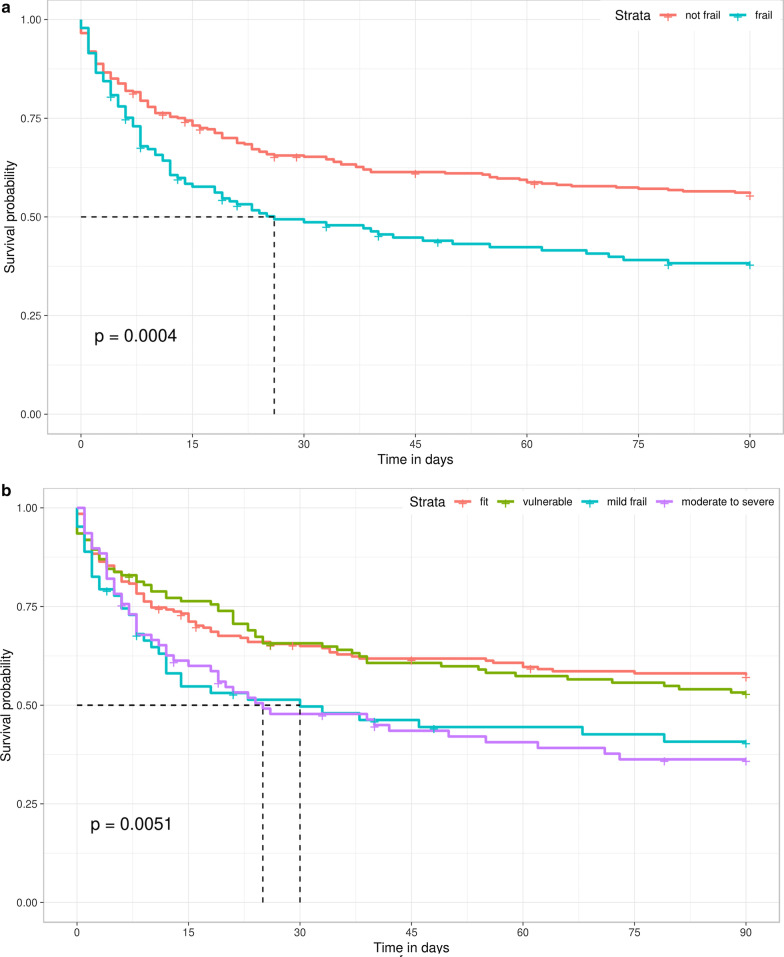
Kaplan–Meier survival curves stratified by baseline frailty status in the OPTIMAL-AKI cohort study. **a** Stratified by binary classification of frail (clinical frailty scale (CFS): 5–8) or not frail (CFS: 1–4). **b** Stratified by categorical classification of frailty status defined as fit (CFS: 1–3), vulnerable (CFS: 4), mild frailty (CFS: 5–6) and moderate-to-severe frailty (CFS: 7–8)

At 90 days, 183 (40%) patients had died. Patients with frailty had greater risk of 90-day mortality compared to those not frail (59% [*n* = 83] vs. 31% [*n* = 100], HR 1.62; CI 1.24–2.13, p < 0.001; Fig. 2), and this difference was observed up to 12 months of follow-up (Additional file 1: Figure S2). After covariate adjustment, frailty was independently associated with 90-day mortality (adjusted HR 1.49; 95% CI 1.11–2.01, *p* = 0.008; Table 2). Complete case analysis yielded similar results (Table S3). However, the addition of frailty status to the model (AUC 0.672; CI 0.622–0.722) did not result in a substantial net reclassification improvement (NRI 0.068; 95% CI − 0.026; 0.162).

**Table 2:** Multivariable Cox regression model for 90-day mortality in the OPTIMAL-AKI cohort

Variables	Crude hazard ratio (95% CI)	*p* Value	Adjusted hazard ratio (95% CI)	*p* Value
Frailty	1.61 (1.23, 2.10)	< 0.001	1.49 (1.11, 2.01)	0.008
Age	1.03 (1.01, 1.04)	0.003	1.02 (1.01, 1.04)	0.012
Sex (female)	0.92 (0.72, 1.19)	0.533	0.92 (0.71, 1.20)	0.560
Charlson score	1.04 (0.99, 1.10)	0.136	1.04 (0.98, 1.10)	0.180
Baseline eGFR per 10 ml/min	1.02 (0.98, 1.06)	0.294	1.03 (0.98, 1.07)	0.260
Peak Serum Creatinine per 50 umol/L	0.97 (0.94, 0.996)	0.026	0.98 (0.95, 1.01)	0.120
SOFA score	1.07 (1.03, 1.10)	< 0.001	1.05 (1.02 1.10)	0.004
APACHE II score	1.04 (1.02, 1.05)	< 0.001	1.06 (1.03, 1.08)	< 0.001
APACHE II score * time^a^	NA	NA	0.998 (0.998, 0.999)	< 0.001

^a^We detected non-proportional hazards for APACHE II scores (non-proportional across time) and accounted for this non-proportionality by modelling the interaction between APACHE II score and time**.**

APACHE II: acute physiology and chronic health evaluation II, eGFR: estimated glomerular filtration rate, SOFA: sequential organ failure assessment

CFS score stratification showed that moderate-to-severe frailty was independently associated with 90-day mortality (adjusted OR 1.91; CI 1.08–3.40, *p* = 0.027) (Additional file 1: Table S4). The addition of the frailty status in this model also did not significantly improve risk prediction (NRI 0.076; CI − 0.016; 0.171]).

### Frailty status at 6 months and 12 months

At 6 months of follow-up, 243 (53%) patients were alive and had CFS scores captured. Among these, 68 (28%) were frail including 39 (57%) patients who were not classified as frail at baseline (Fig. 1). When stratified by frailty severity category, 66 (27%) patients had a worse CFS score at 6 months, while 29 (12%) has an improved CFS score (Additional file 1: Table S5).

At 12 months of follow-up, 216 (47%) patients were alive and had CFS scores captured. Among these, 58 (27%) were considered frail. Between 6 and 12 months, 10 patients (6.0% of patients considered not frail at 6 months) became newly frail while 9 patients (13.2% of patients considered frail at 6 months) transitioned to not frail from previously being classified as frail. When stratified by frailty category, 27 (13%) patients declined to a worse state, while 23 (11%) patients improved (Additional file 1: Table S6).

Among patient characteristics at baseline, age was associated with a deterioration in CFS score at 6 months of follow-up after adjustment (OR 1.08; 95% CI 1.03–1.13, *p* = 0.003). Also, patients with a low CFS score at baseline had a greater likelihood of experiencing a deterioration of frailty status (OR 0.70; 95% CI 0.52–0.94, *p* = 0.019) (Table 3). No other variables were significantly associated with deterioration in frailty status after adjustments, including the receipt of RRT. The resulting model had acceptable discrimination power (AUC 0.70; 95% CI 0.62–0.78) and calibration. Exploratory analysis showed no association between baseline or peak serum creatinine (OR, 0.97; 95% CI 0.81; 1.17; *p* = 0.765 and OR, 1.02; 95% CI 0.97; 1.07; *p* = 0.478, respectively). No associations were found between the candidate variables and a deterioration of frailty status from baseline to 12 months (Additional file 1: Table S7).

**Table 3:** Predictors of deterioration of frailty status* from baseline to 6 months of follow-up

Variables	Univariable		Multivariable	
	OR (CI)	p-value	OR (CI)	p-value
Age	1.04 (1.001–1.09)	0.048	1.08 (1.03–1.13)	0.003
Charlson comorbidity index	0.93 (0.80–1.07)	0.316	0.91 (0.77–1.08)	0.279
APACHE II score	1.01 (0.97–1.05)	0.609	1.01 (0.97–1.06)	0.643
*Pre-hospital disposition*				
Home without assistance	Reference category		Reference category	
Home with assistance	0.53 (0.22–1.33)	0.176	0.94 (0.29–3.01)	0.910
Baseline CFS score	0.78 (0.62–0.99)	0.036	0.70 (0.52–0.94)	0.019
Hospital length of stay	1.01 (0.996–1.10)	0.274	1.01 (0.99–1.02)	0.406
ICU length of stay	0.99 (0.97–1.10)	0.419	0.99 (0.97–1.01)	0.499
Receipt of RRT	0.99 (0.54–1.80)	0.971	0.93 (0.46–1.90)	0.839

*Deterioration of frailty status were defined as any forward transitions from the following categories: Fit (Clinical frailty score (CFS): 1–3), Vulnerable (CFS: 4), Mild Frailty (CFS: 5–6) and Moderate-to-Severe Frailty (CFS: 7–8).

APACHE II: acute physiology and chronic health evaluation II, CFS: clinical frailty scale, CI: confidence interval, ICU: intensive care unit, OR: odds ratio, RRT: renal replacement therapy

### Multistate analysis

A multistate model analysis was performed to determine the influence of individual risk factors on the probability of transitioning between two of the three following states between baseline and 6 months: 1) alive-and-not-frail, 2) alive-and-frail and 3) dead (Additional file 1: Table S8). No factors were associated with transition from alive-and-not frail to alive-and-frail or vice versa. Greater illness acuity was associated with a lower probability of transitioning from alive-and-frail to dead (HR 0.89 CI 0.79–0.997). No factors were associated with transition between states from baseline to 12 months (Additional file 1: Table S9).

## Discussion

We describe the clinical correlates, outcomes and the evolution of frailty status in a large prospective cohort of critically ill older adults with severe AKI. Our data provide new knowledge on implications of frailty status among critically ill patients with severe AKI, a hallmark critical illness severity. Not only was frailty common, present in about 1/3 of patients at baseline, but we also found that frailty portends a higher risk for short and long-term mortality. We showed that clinicians may be less likely to offer RRT to patients perceived as frail. We also found that among survivors not classified as frail at baseline, a substantial proportion of survivors will develop de novo frailty or a worsened frailty status at 6 months and 12 months, while fewer show improvement. We propose that such a transition in functional status may represent a clinically important outcome for patients and/or their families.

Frailty status before critical illness may convey prognostic information and impact care decisions. Most notably, patients with known frailty before critical illness may be perceived differently by clinicians. In addition to clinical characteristics, decision-making at the time where an escalation in the intensity of care is contemplated is likely to be affected by information about previous daily life at home and functional status. We observed that clinicians were less likely to offer RRT for patients with pre-existing frailty and these frail patients were also less likely to receive RRT. However, it must be noted that while baseline frailty status was independently associated with 90-day mortality, the addition of this information to other clinical variables did not result in a meaningful improvement in the prediction of death.

Frailty after critically illness complicated by AKI may be challenging to predict given a “frail state” may be driven by heterogeneous social, cognitive and physical factors that are not readily apparent or measured at ICU admission. While frailty has increasingly been described in general ICU populations [5, 13, 18–24], it has seldom been the focus among critically ill patients with severe AKI. A single-centre analysis of 317 critically ill patients enrolled in a prospective cohort study reported that AKI was associated with a worsened frailty status at 3 and 12 months after hospital discharge compared to those without AKI [9]. Of note, the original study did not specifically focus on an older cohort and excluded patients with pre-existing cognitive deficits. The proportion of survivors with frailty at 12-month follow-up was greater (59%) than observed in our study while the mortality was lower (17%). Similar findings were reported in a recent multicentre observational study of unselected critically ill adults which reported a higher rate of transition to a newly frail state (40%) at 12 months of follow-up, albeit with a much lower mortality rate (25%)[24].

Severe AKI may identify patients more susceptible to prolonged ICU stay [25] and for which rehabilitation may be hampered by the use of RRT [26, 27]. However, neither the duration of ICU stay nor the receipt of RRT were associated with worsened frailty at follow-up in our cohort. Similarly, while it may be evident that a higher burden of baseline chronic disease or the higher acuity of illness should increase the risk of frailty [28], we did not observe this association. Our observations suggest that the risk of developing frailty, a complex multi-dimensional syndrome, cannot be simply extrapolated using simple baseline information. Interestingly, we also report that a substantial proportion of patients showed improvement in their CFS score and frailty status between 6 months and 12 months after critical illness, although the clinical characteristics associated with these transitions remain unclear. From a research perspective, these observations would imply that assessment of frailty status should be extended further than 6 months after critical illness to identify opportunities for intervening to improve outcomes and prevent further functional decline in these patients.

This work has several strengths. Its prospective design minimized recall bias in the assessment of frailty and permitted repeated standardized assessments using a validated scale. Furthermore, data were collected at multiple Canadian centres representative of a diverse critical care population thereby improving generalizability. Also, loss to follow-up (11%) was lower than previous studies [9, 24]. Additionally, this study was the first to study frailty in a population comprised exclusively of AKI patients and report data from an important number of patients with severe AKI. Furthermore, data collection was performed in a relatively short period of time (< 2 years) minimizing the potential impact of changing practice patterns. Finally, this study includes information about the willingness to deliver RRT in the setting of severe AKI which reflects the perceptions of bedside clinicians. The latter is likely to be impacted by information about pre-existing level of functioning prior to hospitalization, which is correlated to frailty.

We recognize some key limitations. Missing frailty assessments at follow-up, although not overly frequent, may have resulted in information bias. Furthermore, while the CFS is a validated tool, it is not the only method to assess frailty and CFS was not compared to other tools such as multi-domain assessments [29]. Most importantly, a single CFS assessment does not provide information about the functional trajectory in the months preceding critical illness which may carry important clinical implications [30, 31]. Inter-rater reliability assessment of CFS was not performed during the study but has previously been shown to be good in similar settings [12]. Second, potentially important socio-demographic and clinical variables that are known to impact frailty status were not collected [32]. Furthermore, most variables capturing illness acuity were recorded at study enrolment while factors occurring later during or after the index hospital stay might impacted frailty status. The lack of serial measures of these time-varying characteristics might explain the lack of associations with frailty status at follow-up. Lastly, the absence of an appropriate control group without severe AKI does not enable us to assess whether frailty develops more frequently in among critically ill survivors with severe AKI.

## Conclusions

In this large multicentre prospective cohort study of older adults with severe AKI, we describe the evolution of frailty status from baseline to 12 months of follow-up and describe that transition to frail state occurs in a substantial proportion of survivors. Although the precise clinical risk factors for this deterioration remain elusive, this information may orient future investigations aimed at better understanding and improving functional outcomes in critically ill patients with severe AKI.

## Supplementary Information

**Additional file 1** Supplementary materials

## Data Availability

The datasets used and/or analysed during the current study are available from the corresponding author on reasonable request.

## Abbreviations

AKI: Acute kidney injury
APACHE II: Acute physiology and chronic health evaluation II
AUC: Area under the curve
CCI: Charlson comorbidity index
CI: Confidence interval
CFS: Clinical frailty scale
eGFR: Estimated glomerular filtration rate
HR: Hazard ratio
ICU: Intensive care unit
NRI: Net reclassification improvement
OR: Odds ratio
SOFA: Sequential organ failure assessment
RRT: Renal replacement therapy
