# Supplementary material for: Frailty status among older critically ill patients with severe acute kidney injury
Source: Crit Care. 2021 Feb 25;25:84. doi: 10.1186/s13054-021-03510-y (PMC7908639; doi:10.1186/s13054-021-03510-y)
Supplement: Supplementary file 1 — Additional file 1 Supplementary materials [file 13054_2021_3510_MOESM1_ESM.docx]

**Supplementary material**

**Figure S1:** Flowchart included participants and available data of frailty

**
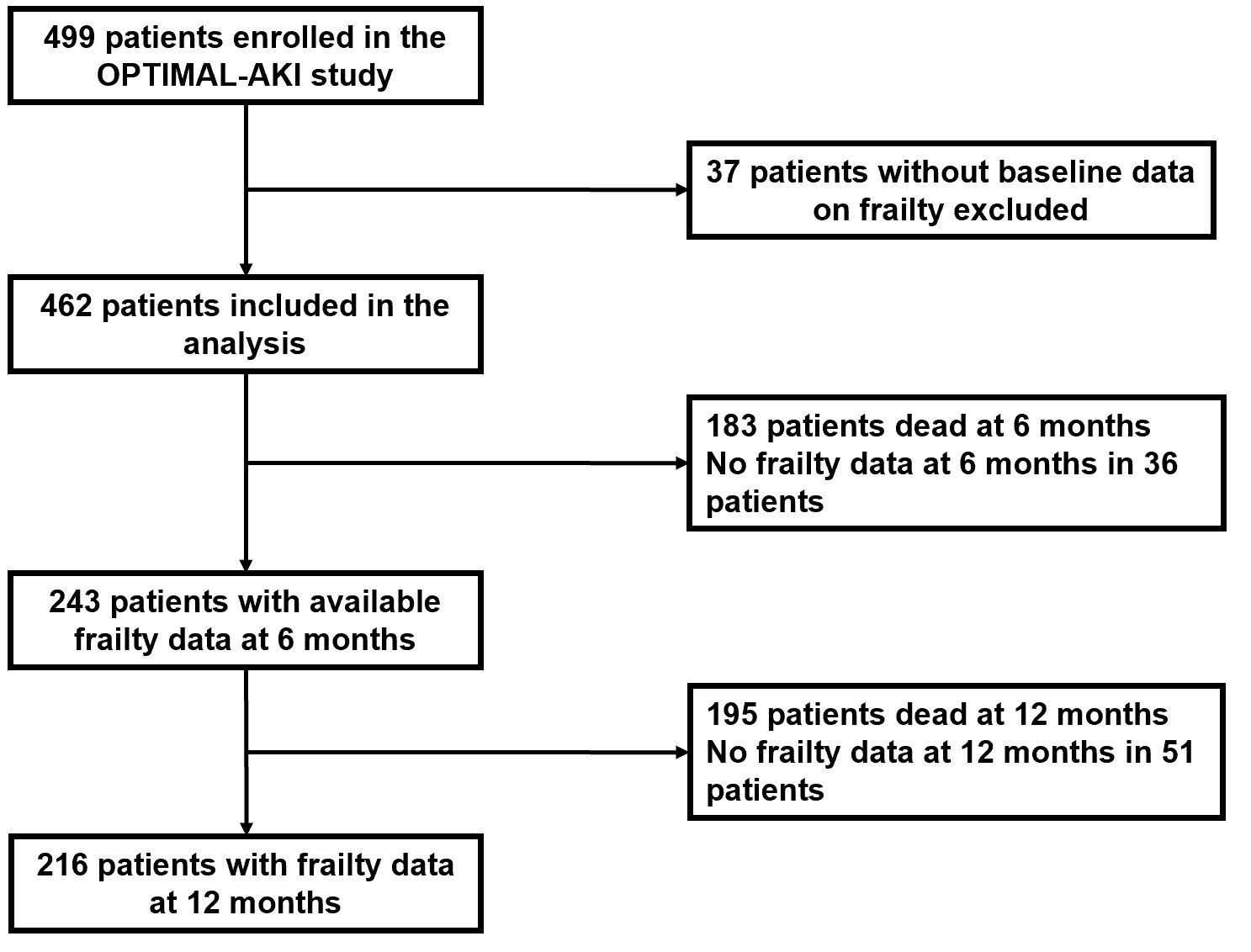
**

**Figure S2** 12-months Kaplan Meir survival curves stratified by baseline frailty status in the OPTIMAL-AKI cohort study. A) Stratified by binary classification of Frail (Clinical Frailty Scale (CFS): 5-8) or Not Frail (CFS: 1-4) B) Stratified by categorical classification of frailty status defined as Fit (CFS: 1-3), Vulnerable (CFS: 4), Mild Frailty (CFS: 5-6) and Moderate-to-Severe Frailty (CFS: 7-8).

**
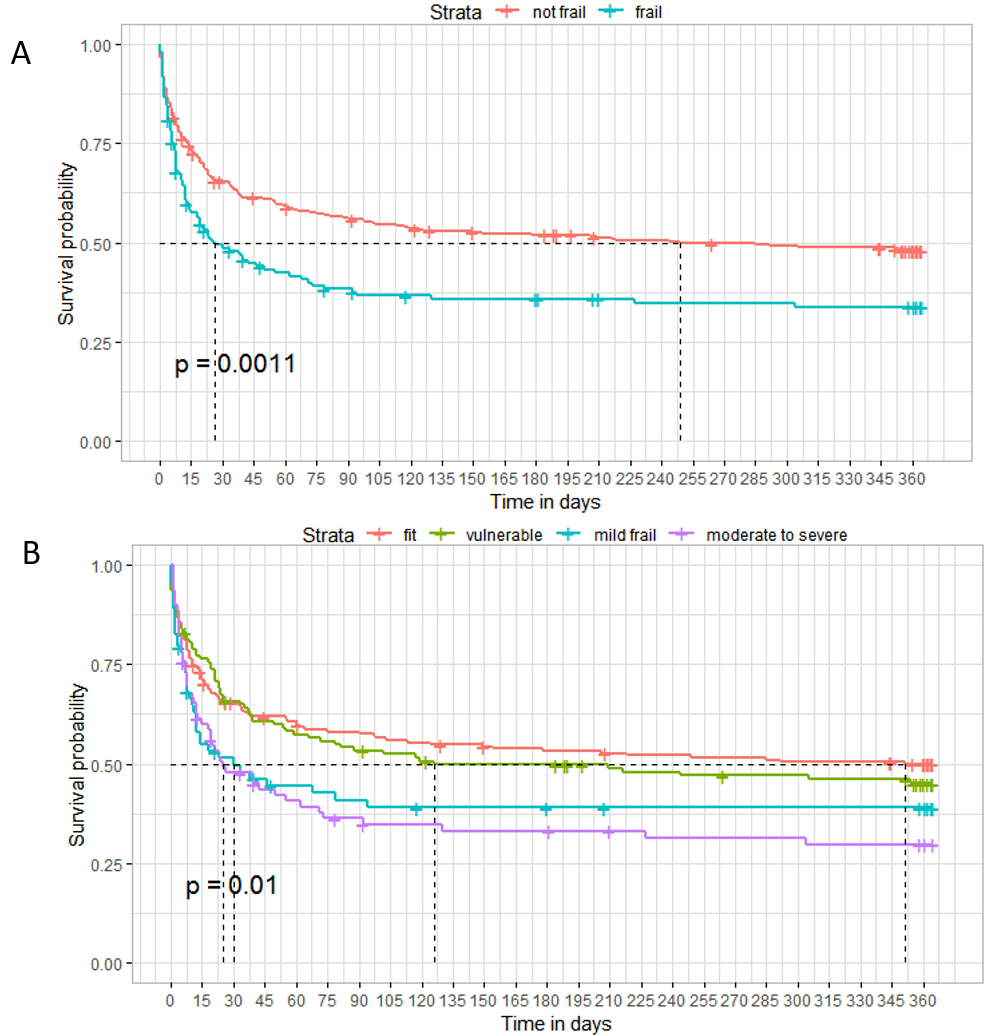
**

**Table S1: Clinical frailty score at baseline, 6 months and 1 year in the studied patients.**

| **Clinical frailty score**  **assessment** | **Timepoint 1:**  **At enrolment** | **Timepoint 2:**  **6 months from enrolment** | **Timepoint 3:**  **1 year from enrolment** |
| --- | --- | --- | --- |
| 1 | 11 | 5 | 9 |
| 2 | 67 | 13 | 20 |
| 3 | 120 | 61 | 36 |
| 4 | 123 | 41 | 39 |
| 5 | 63 | 25 | 23 |
| 6 | 38 | 21 | 20 |
| 7 | 37 | 20 | 14 |
| 8 | 3 | 2 | 0 |
| Median (Q1; Q3) | 4 (3; 5) | 4 (3; 5) | 4 (3; 5) |

**Table S2:** Summary of patient characteristics stratified by frailty status (alternative classification) at baseline.

| **Variable** | **Fit**  198 (42.9%) | **Vulnerable**  123 (26.6%) | **Mild Frailty**  63 (13.6%) | **Severe Frailty**  78 (16.9%) | **p-value** | **missing** |
| --- | --- | --- | --- | --- | --- | --- |
| **Demographic information** | | | | | | |
| Age (mean [SD]), years | 74.0 (6.9) | 74.9 (6.5) | 77.1 (8.0) | 77.6 (8.3) | <0.001 | 0.0 |
| Female sex, (n, %) | 64 (32.3) | 50 (40.7) | 34 (54.0) | 40 (51.3) | 0.003 | 0.0 |
| Charlson score, (median [IQR]) | 2.0 (1.0, 3.8) | 3.0 (2.0, 4.0) | 3.0 (1.0, 5.0) | 3.5 (2.0, 5.0) | <0.001 | 0.2 |
| **Comorbid diseases, (n, %)** |  |  |  |  |  |  |
| Congestive heart failure | 31 (15.7) | 36 (29.5) | 16 (25.4) | 28 (35.9) | 0.001 | 0.2 |
| Chronic obstructive pulmonary disease | 47 (23.7) | 38 (31.1) | 18 (28.6) | 28 (35.9) | 0.193 | 0.2 |
| Connective tissue disease | 10 (5.1) | 6 (4.9) | 6 (9.5) | 5 (6.4) | 0.571 | 0.2 |
| Diabetes mellitus | 61 (30.8) | 62 (50.8) | 22 (34.9) | 37 (47.4) | 0.001 | 0.2 |
| Peripheral vascular disease | 20 (10.1) | 21 (17.2) | 10 (15.9) | 20 (25.6) | 0.012 | 0.2 |
| Any cancer | 43 (21.7) | 20 (16.4) | 9 (14.3) | 17 (21.8) | 0.435 | 0.2 |
| Chronic liver disease | 4 (2.0) | 4 (3.3) | 5 (7.9) | 3 (3.8) | 0.169 | 0.2 |
| **Functional status** | | | | | | |
| Cognition (CSI-D screen), (n, %)* |  |  |  |  |  |  |
| Dementia | 1 (0.5) | 1 (0.9) | 4 (7.0) | 14 (19.2) | <0.001 | 13.0 |
| Impaired - not demented | 9 (4.9) | 19 (17.1) | 17 (29.8) | 27 (37.0) |  |  |
| No impairment | 175 (94.6) | 91 (82.0) | 36 (63.2) | 32 (43.8) |  |  |
| Pre-hospital location |  |  |  |  |  |  |
| Home - independent | 183 (92.4) | 100 (82.0) | 35 (56.5) | 10 (12.8) | <0.001 | 2.8 |
| Home - with assistance | 11 (5.6) | 18 (14.8) | 23 (37.1) | 49 (62.8) |  |  |
| Assisted living | 4 (2.0) | 4 (3.3) | 4 (6.5) | 19 (24.4) |  |  |
| Hospitalized in prior 6 months, (n, %) | 61 (30.8) | 49 (39.8) | 29 (46.0) | 37 (47.4) | 0.027 | 1.8 |
| **Current illness** |  |  |  |  |  |  |
| Primary diagnostic category, (n, %) | | | | | | |
| Cardiovascular | 49 (25.1) | 34 (27.9) | 22 (34.9) | 16 (20.5) | 0.098 | 0.8 |
| Respiratory | 38 (19.5) | 26 (21.3) | 10 (15.9) | 19 (24.4) |  |  |
| Gastrointestinal/hepatic | 32 (16.4) | 10 (8.2) | 2 (3.2) | 10 (12.8) |  |  |
| Metabolic/endocrine | 13 (6.7) | 15 (12.3) | 5 (7.9) | 6 (7.7) |  |  |
| Neurologic | 3 (1.5) | 0 (0.0) | 3 (4.8) | 2 (2.6) |  |  |
| Hematologic/oncologic | 5 (2.6) | 3 (2.5) | 0 (0.0) | 0 (0.0) |  |  |
| Sepsis | 49 (25.1) | 30 (24.6) | 21 (33.3) | 24 (30.8) |  |  |
| Trauma | 6 (3.1) | 4 (3.3) | 0 (0.0) | 1 (1.3) |  |  |
| APACHE II score (mean [SD]) | 28.2 (8.6) | 28.7 (9.1) | 26.5 (8.4) | 28.8 (8.8) | 0.406 | 3.6 |
| SOFA score (mean [SD]) | 11.0 (4.5) | 10.7 (4.1) | 9.5 (4.0) | 10.1 (4.0) | 0.058 | 4.0 |
| Mechanical ventilation (n, %) | 128 (65.6) | 78 (68.4) | 35 (58.3) | 52 (67.5) | 0.586 | 3.4 |
| Vasoactive support (n, %) | 135 (69.2) | 77 (67.5) | 42 (70.0) | 47 (61.0) | 0.593 | 3.4 |
| Blood transfusion (n, %) | 33 (16.9) | 31 (27.2) | 7 (11.7) | 10 (13.0) | 0.023 | 3.4 |
| TPN (n, %) | 12 (6.2) | 4 (3.5) | 2 (3.3) | 2 (2.6) | 0.503 | 3.4 |
| Baseline serum creatinine, (median [IQR]) | 95.0 (73.0, 138.0) | 99.5 (68.8, 147.8) | 97.0 (71.0, 140.5) | 97.0 (82.2, 127.5) | 0.948 | 0.6 |
| Baseline eGFR, (mean [SD]) | 56.1 (30.5) | 55.1 (31.7) | 55.2 (28.6) | 56.8 (34.2) | 0.979 | 0.6 |
| Worst KDIGO AKI stage, (n, %) |  |  |  |  |  |  |
| Stage 2 | 38 (19.3) | 20 (16.3) | 12 (19.0) | 16 (20.5) | 0.873 | 0.2 |
| Stage 3 | 159 (80.7) | 103 (83.7) | 51 (81.0) | 62 (79.5) |  |  |
| Receipt of RRT |  |  |  |  |  |  |
| Peak serum creatinine, (median [IQR]) | 359.5 (252.2, 491.5) | 345.0 (228.0, 495.5) | 270.0 (205.0, 407.0) | 312.0 (213.5, 481.8) | 0.027 | 0.0 |
| Peak BUN, (median [IQR]) | 28.0 (18.1, 39.6) | 27.7 (20.6, 34.8) | 23.0 (16.0, 30.3) | 26.0 (17.0, 35.4) | 0.042 | 2.4 |
| GOC documentations/discussion at ICU admission (n, %) | 136 (68.7) | 78 (63.4) | 39 (61.9) | 52 (66.7) | 0.685 | 0.4 |
| Physician’s willingness to offer RRT (n, %) | 159 (80.3) | 85 (69.1) | 39 (61.9) | 50 (64.1) | 0.005 | 0.0 |

Abbreviations: AKI: acute kidney injury, APACHE II: Acute Physiology And Chronic Health Evaluation II, eGFR: estimated glomerular filtration rate, KDIGO: Acute Physiology And Chronic Health Evaluation II SD: standard deviation, SOFA: sequential organ failure assessment, RRT: renal replacement therapy

**Table S3:** Multivariable Cox regression model for 90-day mortality in the OPTIMAL-AKI cohort. (Complete case analysis without multiple imputation for missing variables)

| **Covariates** | **Crude Odds Ratio**  **(95% CI)** | **P-value** | **Adjusted Hazard Ratio (95% CI)** | **P-value** |
| --- | --- | --- | --- | --- |
| CFS (frail) | 1.624  ( 1.239, 2.130 ) | 0.0005 | 1.453  ( 1.071 , 1.973 ) | 0.0165 |
| Age | 1.026  ( 1.009, 1.043 ) | 0.0030 | 1.024  ( 1.004 , 1.044 ) | 0.0164 |
| Sex (female) | 1.083  ( 0.843, 1.393 ) | 0.5324 | 1.139  ( 0.859 , 1.510 ) | 0.3667 |
| Charlson Score | 1.040  ( 0.987, 1.097 ) | 0.1438 | 1.055  ( 0.995 , 1.119 ) | 0.0726 |
| Baseline eGFR per 10 ml/min | 1.021  ( 0.982, 1.061 ) | 0.3014 | 1.027  ( 0.981 , 1.074 ) | 0.2532 |
| Peak Serum Creatinine per 50 umol/L | 0.968  ( 0.941, 0.996 ) | 0.0262 | 0.979  ( 0.945 , 1.011 ) | 0.2023 |
| SOFA Score | 1.063  ( 1.031, 1.096 ) | 0.0000 | 1.062  ( 1.021 , 1.105 ) | 0.0027 |
| APACHE II score | 1.037  ( 1.022, 1.053 ) | 0.0000 | 1.055  ( 1.031 , 1.079 ) | 0.0000 |
| APACHE II score * time | NA | NA | 0.998  ( 0.998 , 0.999 ) | 0.0000 |

Abbreviations: APACHE II: Acute Physiology And Chronic Health Evaluation II, CFS: Clinical Frailty Scale, CI: confidence interval, ICU: Intensive Care Unit, OR: Odds ratio, RRT: Renal Replacement Therapy.

**Table S4:** Multivariable logistic regression model for 90-day mortality in the OPTIMAL-AKI cohort including the 4-categories frailty status.

| **Covariates** | **Crude Odds Ratio**  **(95% CI)** | **P-value** | **Adjusted Odds Ratio**  **(95% CI)** | **P-value** |
| --- | --- | --- | --- | --- |
| CFS (vulnerable) | 1.1799  ( 0.7399, 1.8816 ) | 0.4872 | 1.1553  ( 0.7243 , 1.8429 ) | 0.5448 |
| CFS (mild frail) | 1.7926  ( 0.9901 , 3.2456 ) | 0.0540 | 1.7377  ( 0.9357 , 3.2271 ) | 0.0808 |
| CFS (moderate to severe) | 2.1073  ( 1.2184 , 3.6447 ) | 0.0077 | 1.9148  ( 1.0772 , 3.4039 ) | 0.0273 |
| Age | 1.026 (1.009, 1.04) | 0.0030 | 1.0372  ( 1.0094 , 1.0658 ) | 0.0088 |
| Sex (female) | 1.083 (0.843, 1.39) | 0.5320 | 0.9257  ( 0.6264 , 1.3678 ) | 0.6983 |
| Charlson Score | 1.040 (0.987, 1.10) | 0.1440 | 1.031  ( 0.9474 , 1.1219 ) | 0.4800 |
| Baseline eGFR | 1.026 (0.982, 1.061) | 0.3010 | 1.0041  ( 0.9976 , 1.0106 ) | 0.2217 |
| Peak Serum Creatinine | 0.968 (0.941, 0.996) | 0.0262 | 0.9994  ( 0.9986 , 1.0002 ) | 0.1375 |
| SOFA Score | 1.063 (1.031, 1.096) | 0.0000 | 1.0633  ( 1.0095 , 1.12 ) | 0.0209 |
| APACHE II score | 1.037 (1.022, 1.053) | 0.0000 | 1.0297  ( 1.005 , 1.055 ) | 0.0187 |

Abbreviations: APACHE II: Acute Physiology And Chronic Health Evaluation II, CFS: Clinical Frailty Scale, CI: confidence interval, ICU: Intensive Care Unit, OR: Odds ratio, RRT: Renal Replacement Therapy.

**Table S5: Clinical frailty score at baseline and after 6 months of follow-up. Red indicates a deterioration of frailty status (n=66) while green indicate an improvement (n=29).**

|  | **6 months of follow-up** | | | | |
| --- | --- | --- | --- | --- | --- |
| **Baseline** |  | **Fit** | **Vulnerable** | **Mild frailty** | **Moderate-to-severe**  **frailty** |
|  | **Fit** | 112 | 21 | 9 | 10 |
|  | **Vulnerable** | 18 | 15 | 10 | 10 |
|  | **Mild frailty** | 3 | 5 | 4 | 6 |
|  | **Severe frailty** | 1 | 0 | 2 | 17 |

**Table S6: Clinical frailty score at 6 months and 12 months of follow-up. Red indicates a deterioration of frailty status (n=27) while green indicate an improvement (n=23).**

|  | **12 months of follow-up** | | | | |
| --- | --- | --- | --- | --- | --- |
| **6 months of follow-up** |  | **Fit** | **Vulnerable** | **Mild frailty** | **Moderate-to-severe**  **frailty** |
|  | **Fit** | 108 | 13 | 0 | 1 |
|  | **Vulnerable** | 7 | 21 | 6 | 3 |
|  | **Mild frailty** | 3 | 3 | 10 | 4 |
|  | **Severe frailty** | 1 | 2 | 7 | 27 |

**Table S7 Predictors of deterioration of frailty status* from baseline to 12 months of follow-up.**

| **Variables** | **Univariable** | | **Multivariable** | |
| --- | --- | --- | --- | --- |
|  | **OR (CI)** | **p-value** | **OR (CI)** | **p-value** |
| **Age** | 1.015 (0.971 – 1.061) | 0.5151 | 1.044 (0.991 – 1.099) | 0.1077 |
| **Charlson comorbidity index** | 1.020 (0.881 – 1.182) | 0.7904 | 0.988 (0.839 – 1.164) | 0.8871 |
| **APACHE II score** | 0.993 (0.955 – 1.032) | 0.7219 | 0.983 (0.941 – 1.027) | 0.4458 |
| **Pre-hospital disposition** |  |  |  |  |
| **Home without assistance** | Reference category |  | Reference category |  |
| **Home with assistance** | 0.607 (0.239 – 1.544) | 0.2944 | 0.846 (0.266 – 2.691) | 0.7766 |
| **Baseline CFS Score** | 0.814 (0.637 – 1.041) | 0.1005 | 0.789 (0.577 – 1.077) | 0.1357 |
| **Hospital length of stay** | 1.002 (0.993 – 1.010) | 0.6767 | 1.003 (0.994 – 1.013) | 0.5360 |
| **ICU length of stay** | 0.978 (0.947 – 1.010) | 0.1816 | 0.977 (0.940 – 1.016) | 0.2405 |
| **Receipt of RRT** | 1.381 (0.724 – 2.634) | 0.3272 | 1.645 (0.762 – 3.554) | 0.2053 |

* Deterioration of frailty status were defined as any forward transitions from the following categories: Fit (Clinical frailty score (CFS): 1-3), Vulnerable (CFS: 4), Mild Frailty (CFS: 5-6) and Moderate-to-Severe Frailty (CFS: 7-8). Abbreviations: APACHE II: Acute Physiology And Chronic Health Evaluation II, CFS: Clinical Frailty Scale, CI: confidence interval, ICU: Intensive Care Unit, OR: Odds ratio, RRT: Renal Replacement Therapy.

**Table S8 Multistate model to determine the association between risk factors at baseline and transitions between the following states at 6 months of follow-up.**

| **Variables** | **Transition from**  **alive and not frail to alive and frail (1 to 2)** | **Transition from**  **alive and frail to alive and not frail (2 to 1)** | **Transition from**  **alive and not frail to dead (1 to 3)** | **Transition from**  **Alive and frail to dead (2 to 3)** |
| --- | --- | --- | --- | --- |
| **Age** | 1.02 (0.96 – 1.09) | 0.90 (0.78 – 1.04) | 1.18 (0.78 – 1.77) | 0.94 (0.79 – 1.11) |
| **Charlson comorbidity score** | 0.98 (0.76 – 1.23) | 1.30 (0.83 – 2.02) | 1.14 (0.85 – 1.53) | 1.08 (0.74 – 1.59) |
| **APACHE II score** | 1.006 (0.960 – 1.054) | 0.912 (0.808 – 1.029) | 1.048 (0.926 – 1.185) | 0.889 (0.793 – 0.997) |
| **Pre-hospital disposition** |  |  |  |  |
| **Home without assistance** | Reference category | | | |
| **Home with assistance or**  **in assisted living facility** | 0.97 (0.25 – 3.80) | 0.15 (0.01 – 2.94) | 3.87 (0.11 – 143.16) | 0.86 (0.07 – 10.25) |
| **Hospital length of stay** | 0.998 (0.989 – 1.008) | 0.932 (0.860 – 1.011) | 0.991 (0.952 – 1.031) | 1.000 (0.975 – 1.025) |
| **ICU length of stay** | 0.993 (0.965 – 1.021) | 0.903 (0.712 – 1.145) | 1.024 (0.958 – 1.096) | 1.004 (0.975 – 1.034) |
| **Receipt of RRT** | 0.88 (0.28 – 2.80) | 0.50 (0.06 – 4.37) | 0.44 (0.06 – 3.11) | 2.50 (0.35 – 17.93) |

Legend: Associations are presented as Hazard Ratio (HR) with 95% confidence intervals. Abbreviations: APACHE II: Acute Physiology And Chronic Health Evaluation II, CFS: Clinical Frailty Scale, CI: confidence interval, ICU: Intensive Care Unit, OR: Odds ratio, RRT: Renal Replacement Therapy.

**Table S9 Multistate model to determine the association between risk factors at baseline and transitions between the following states at 12 months of follow-up.**

| **Variables** | **Transition from**  **alive and not frail to alive and frail (1 to 2)** | **Transition from**  **alive and frail to alive and not frail (2 to 1)** | **Transition from**  **alive and not frail to dead (1 to 3)** | **Transition from**  **Alive and frail to dead (2 to 3)** |
| --- | --- | --- | --- | --- |
| **Age** | 0.978 (0.872 – 1.097) | 0.881 (0.739 – 1.051) | 1.060 (0.910 – 1.234) | 0.995 (0.834 – 1.186) |
| **Charlson comorbidity score** | 1.062 (0.847 – 1.330) | 1.483 (0.864 – 2.544) | 1.036 (0.794 – 1.351) | 1.111 (0.733 – 1.684) |
| **APACHE II score** | 0.985 (0.886 – 1.096) | 0.869 (0.714 – 1.058) | 0.995 (0.888 – 1.114) | 0.918 (0.802 – 1.051) |
| **Pre-hospital disposition** |  |  |  |  |
| **Home without assistance** | Reference category | | | |
| **Home with assistance or**  **in assisted living facility** | 1.952 (0.459 – 8.298) | 0.162 (0.016 – 1.631) | 2.110 (0.441 – 10.090) | 1.559 (0.125 – 19.449) |
| **Hospital length of stay** | 1.001 (0.992 – 1.010) | 0.913 (0.781 – 1.068) | 0.999 (0.985 – 1.013) | 0.998 (0.969 – 1.028) |
| **ICU length of stay** | 0.980 (0.926 – 1.037) | 0.902 (0.505 – 1.613) | 1.014 (0.974 – 1.056) | 0.994 (0.934 – 1.057) |
| **Receipt of RRT** | 1.485 (0.449 – 4.907) | 0.523 (0.036 – 7.534) | 1.024 (0.202 – 5.181) | 1.514 (0.390 – 5.882) |

Legend: Associations are presented as Hazard Ratio (HR) with 95% confidence intervals. Abbreviations: APACHE II: Acute Physiology And Chronic Health Evaluation II, CFS: Clinical Frailty Scale, CI: confidence interval, ICU: Intensive Care Unit, OR: Odds ratio, RRT: Renal Replacement Therapy.
